# AAscan, PCRdesign and MutantChecker: A Suite of Programs for Primer Design and Sequence Analysis for High-Throughput Scanning Mutagenesis

**DOI:** 10.1371/journal.pone.0078878

**Published:** 2013-10-30

**Authors:** Dawei Sun, Martin K. Ostermaier, Franziska M. Heydenreich, Daniel Mayer, Rolf Jaussi, Joerg Standfuss, Dmitry B. Veprintsev

**Affiliations:** Laboratory of Biomolecular Research, Paul Scherrer Institut, Villigen, Switzerland and Department of Biology, ETH Zurich, Zurich, Switzerland; CSIR-Institute of Microbial Technology, India

## Abstract

Scanning mutagenesis is a powerful protein engineering technique used to study protein structure-function relationship, map binding sites and design more stable proteins or proteins with altered properties. One of the time-consuming tasks encountered in application of this technique is the design of primers for site-directed mutagenesis. Here we present an open-source multi-platform software AAscan developed to design primers for this task according to a set of empirical rules such as melting temperature, overall length, length of overlap regions, and presence of GC clamps at the 3’ end, for any desired substitution. We also describe additional software tools which are used to analyse a large number of sequencing results for the presence of desired mutations, as well as related software to design primers for ligation independent cloning. We have used AAscan software to design primers to make over 700 mutants, with a success rate of over 80%. We hope that the open-source nature of our software and ready availability of freeware tools used for its development will facilitate its adaptation and further development. The software is distributed under GPLv3 licence and is available at http://www.psi.ch/lbr/aascan.

## Introduction

Site-directed mutagenesis is a cornerstone of protein engineering. It was first used to study the function of the catalytic residues in the tyrosyl-tRNA synthetase [1]. Surface scanning using site-directed mutagenesis, a systematic substitution of protein residues by other amino acids, notably alanine, was first employed to characterise the binding site of the C1q, a component of an immune response system, on the surface of the Fc domain of an antibody [2]. The term alanine scanning was coined by Jim Wells in the mapping of the human growth hormone interaction surface with the corresponding receptor [3]. Alanine scanning has become an indispensable technique to study structure-function relationship in proteins, to obtain structural information about their folding intermediates as well as to engineer them for altered stability or functionality [4], [5], [6], [7], [8], [9].

In order to introduce a mutation into a recombinantly produced protein, its coding DNA sequence is modified by using mutagenesis primers in a polymerase chain reaction (PCR). Numerous software solutions to primer design task already exist that design mutagenesis primers according to a set of empirical rules and some take the complexity of the template into account. However, when it comes to designing several hundred primers for scanning mutagenesis, no convenient solution is openly accessible. Here, we present the AAscan software to automatically design batches of primers to perform a scanning mutagenesis of a whole protein. We used this software to make several hundreds of alanine mutants of Gαi1 and arrestin-1.

## Results

### Mutagenesis strategy and workflow

**Figure 1:**
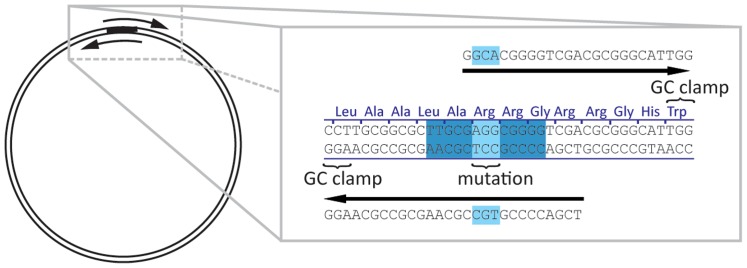
Mutagenesis by overlapping PCR reaction.

A convenient way of performing site-directed mutagenesis is an adaptation [10] of the original ligation-independent cloning protocol [11]. The target plasmid is amplified with two primers which contain the mutation site **(Figure 1)** leading to a linear PCR product with short identical sequence at both ends. Transformation into *E. coli* Mach1, NovaBlue or TG1 strains leads to end repair which restores a circular plasmid containing the introduced mutation [12], [13]. While the exact mechanism of this reaction is not well understood, from our experience the mentioned above cell strains are more efficient than other cell strains commonly used for DNA manipulation. The repair requires a minimal primer overlap of 13 bp. The mutation site could be located anywhere in the pair of primers and not necessarily in the overlap region, however, certain limitations are discussed below. Unlike the PCR product, the original template is methylated. This allows digestion of the template with DpnI in order to minimise the background. Alternatively, the PCR template may be methylated enzymatically at CpG dinucleotides and is eliminated by certain strains of E. coli that contain wt McrBC restriction system [14]. After transformation and plating with appropriate antibiotics, several single colonies are sent for sequencing as bacterial slabs on a 96-well plate. If mutagenesis was not successful after three attempts, alternative methods were used to generate the remaining mutants.

### Primer design by AAscan

**Figure 2:**
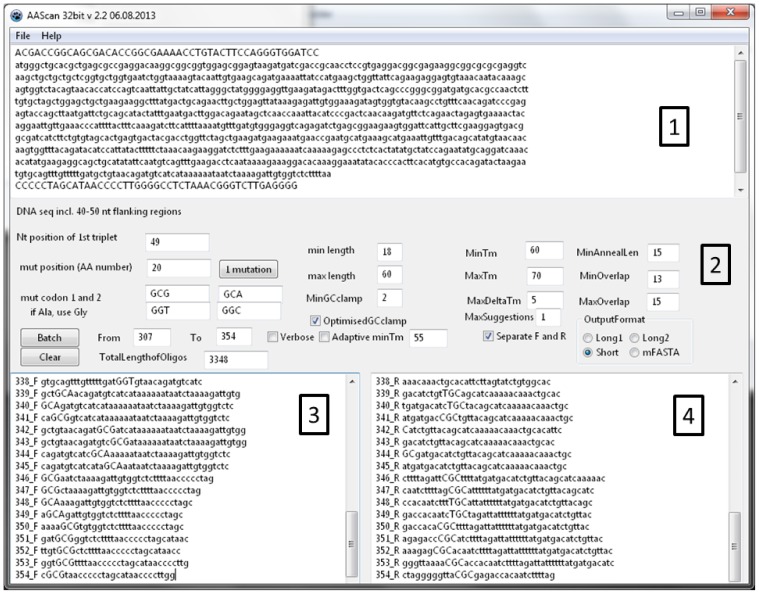
AAscan software interface. (1) Input text box for reference sequence. (2) Options for primer design. (3) Output window containg primer forward and (4) reverse primers.

The software interface **(Figure 2)** includes a text box for entering the template DNA sequence, the choice of codons to be used for mutagenesis, fields to define the region of the protein sequence to be mutated, various options used in primer design described above and, finally, different options for the output data format.

The template sequence for scanning mutagenesis include the flanking regions of 50-60 bp (but at least with a length equivalent to the number of bp given in the field “**max length**”) as the primers may anneal outside of the protein coding region, if the position to be mutated is close to the protein termini. The flanking regions are shown in capital letters in **Figure 2**.

The position of the 1^st^ nucleotide in the protein coding sequence needs to be specified so that the software can convert the amino acid position to nucleotide coordinates. For example, if the 48 bp of the vector upstream of the protein coding sequence are included, the position of A from the first ATG is 49.

Two different codons for mutagenesis need to be specified for the desired substitution, and the program will choose the one that introduces the least number of mismatches. If the template codon is already encoding an alanine, the codon will be mutated to another amino acid, for example glycine. Two alternative codons choices are provided. Of course, the mutations can be designed for any desired amino acid, not only alanine. If only one codon encodes a particular amino acid, or use of a particular codon is preferred, the same codon needs to be entered. If more than two codons encode a particular amino acid, the two preferred codons are selected based on the expression organism and goals of the project.

To design the primers, either a single amino acid position or a range needs to be specified.

AAscan designs the shortest primers with a length in between the “min length” and “max length” entered by the user. The melting temperature (*T*m) is designed to be as close to the “min*T*m” as possible, not exceeding the “max*T*m”. The maximal difference between melting temperatures of forward and reverse primers may not exceed the value given in “MaxDelta*T*m”.

Tm is calculated according to the following formula [15]:

T_m_  =  64.9°C + 41°C x (number of G’s and C’s in the primer – 16.4)/length of the primer (1)

Two melting temperatures are reported. The first (*T*m) is relevant for the initial cycles of the PCR when the primer anneals to the original template DNA, with mismatches in the mutation site. The second *T*m value (*T*m_full_) is relevant for the later stages of the PCR, when sufficient product was already amplified and the full length sequence of the primer anneals to the newly synthesised template without mismatches. The increased stability of primers may lead to a change in the efficiency of the PCR reaction.

In addition, several further parameters are taken into account:

“MinAnnealLen” is the minimal distance from the mutation codon to the 3’ end of the primer, and in our experience it should be at least 15bp.

“minGCclamp” is the minimal number of G or C bases at the 3′ end of the primer. The “minGCclamp” can be set to 0 if the GC clamp is not required.. If the checkbox **“**OptimisedGCclamp**”** is selected, “OptimisedGCclamp**”** score is calculated according to the rules formulated in [16]. Depending on the combination of the last three nucleotides of the 3’ end of the primer, score is assigned as follows: [GC] [GC] [GC] = 0; [ATGC] [ATGC] [AT] = 1; [ATGC] [AT] [GC] = 2; and [AT] [GC] [GC] = 3. The score of 0 corresponds to the worst GC clamp and 3 to the best, respectively. In this context, [ATGC] means any nucleotide, [AT] means A or T, and [GC] means G or C, respectively.

“MinOverlap” and “MaxOverlap” is the length of the overlap sequence between the ends of the resulting PCR fragment. The default range is 13 to 15 bp. If the overlap is too short (<11 bp), this will result in decreased efficiency of end repair [17], while too long overlaps may lead to undesired self-annealing of primers, formation of wrong PCR products and decrease in overall PCR efficiency.

The number of primer pairs designed for the mutation of every specified amino acid position is defined by the filed “Maxsuggestion”. When generating primers in batch mode, this parameter is generally set to ”1”.

The “Adaptive Tm” option can be used when for some positions in the gene no primers can be generated within the constraints of maximal length and minimal *T*m. For these positions only, the program will decrease the “minTm” value by 1°C until it can generate a primer pair satisfying all constraints. However, a better alternative is to increase the maximum length of the primer.

Different output formats of the primer design are applicable. The “Long1” format provides the sequence of the suggested primer on the coding strand, with capitalised mutation site and introduced mutations denoted by an “X”, a list of parameters describing its properties such as length, Tm excluding mismatches, Tm of the full length primer, GC clamp score, annealing length from the mutation site to the 3’ end, and the actual sequence of the primer to be ordered, reverse-complemented if needed. “Long2” format provides the same information in tabular format convenient for import into spreadsheets. “Short” generates a list of oligo names and sequences (reverse-complemented if needed) to be ordered – convenient for generating orders. “FASTA” generates a FASTA formatted list of primers.

“Separate F and R” checkbox creates two separate lists of forward and reverse oligonucleotides which can be useful for ordering.

“Verbose” activates additional information printout which may helpful for primer design.

The results of the primer design from one or both output text boxes can be copied to the clipboard and pasted in the order form.

“File/Open” and “File/Save” menu items allow opening and saving of the projects (sequences and options). The data are saved in binary format and manual editing of the data file is not supported.

### MutantChecker: sequencing results analysis

**Figure 3:**
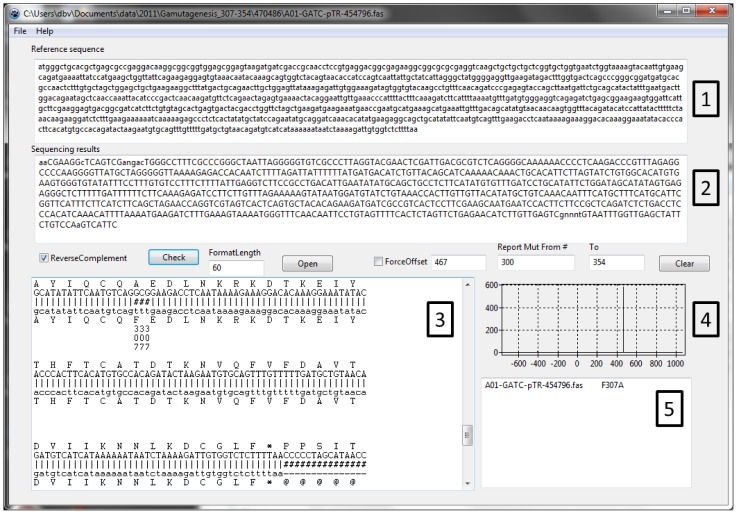
Mutant Checker interface. (1) Input text box for reference sequence, starting with the beginning of the expressed sequence (first ATG). (2) Input text box for sequencing results. The sequence is reverse completented for the analyis if the “ReverseComplement” checkbox is activated. (3) Output windows of aligned sequences, DNA to protein translation of both reference sequence showing identified mutations and their position, based on the reference sequence. (4) Graphical output of the aligment score function vs offset between to sequences. A single peak indicate only one possible aligment, while multiple peaks indicate several alternative aligments and non-productive clone. (5) Identified mutations, within the specified region.



The amount of sequencing results needed to be analysed stimulated the development of a software to align and identify mutations semi-automatically **(Figure 3)**. Mutant checker is a software designed to align and identify mutations semi-automatically which facilitates analysis of large numbers of sequencing results. It aligns the sequencing results to the reference sequence using an empirical likelihood function. This a number of perfect matches in a sliding track of 7 nucleotides along the whole length of reference sequence. This function (Eq. 1) does not allow gaps, as we are looking only for mutations, and not deletions or insertions.

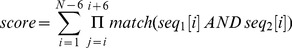
(1)


For every possible offset between two sequences to be compared, an overlap region is copied into *seq*_1_ and *seq*_2_. The length of the overlap region is *N*. The match function compares if the nucleotides *seq*_1_ [i] and *seq*_2_ [i] at position *i* are the same (result = 1) or different (result = 0). By combining the match function over a sliding window of 7 nucleotides, the software calculates if all 7 nucleotides are the same. The sliding window is then moved by one nucleotide, and calculations are repeated. The total score for the alignment with a given offset is a sum of individual scores. The maximal score corresponds to the best alignment. The function has significant differentiating power between right and wrong alignments.

If the sequencing results contain the desired mutation and no additional rearrangements, then there will be only one possible alignment. On the other hand, if during recombination event in the cell part of the sequence was duplicated, there will be two or more possible alignment positions reported on a graph.

The MutantChecker can also reverse complement the sequence if the sequencing was done with reverse primers, for mutations close to the C-terminus of the protein. The software can also process batches of sequences which dramatically speeds up the analysis of sequencing results.

### PCR cloning primer design software

Seamless cloning has gained acceptance as a very convenient cloning method which allows seamless integration of the desired DNA sequences into a vector [12], [18]. Versions of this method also allow deletion or replacement of a part of a sequence with a single PCR reaction. At the core of the method is a PCR amplification of the vector backbone and, in a separate reaction, amplification of the desired insert, so that the ends have identical sequences overlapping by about 15 bp. During the PCR stage it is also possible to have additional sequences included in the primer, for example a coding sequence for a protease restriction site.

**Figure 4:**
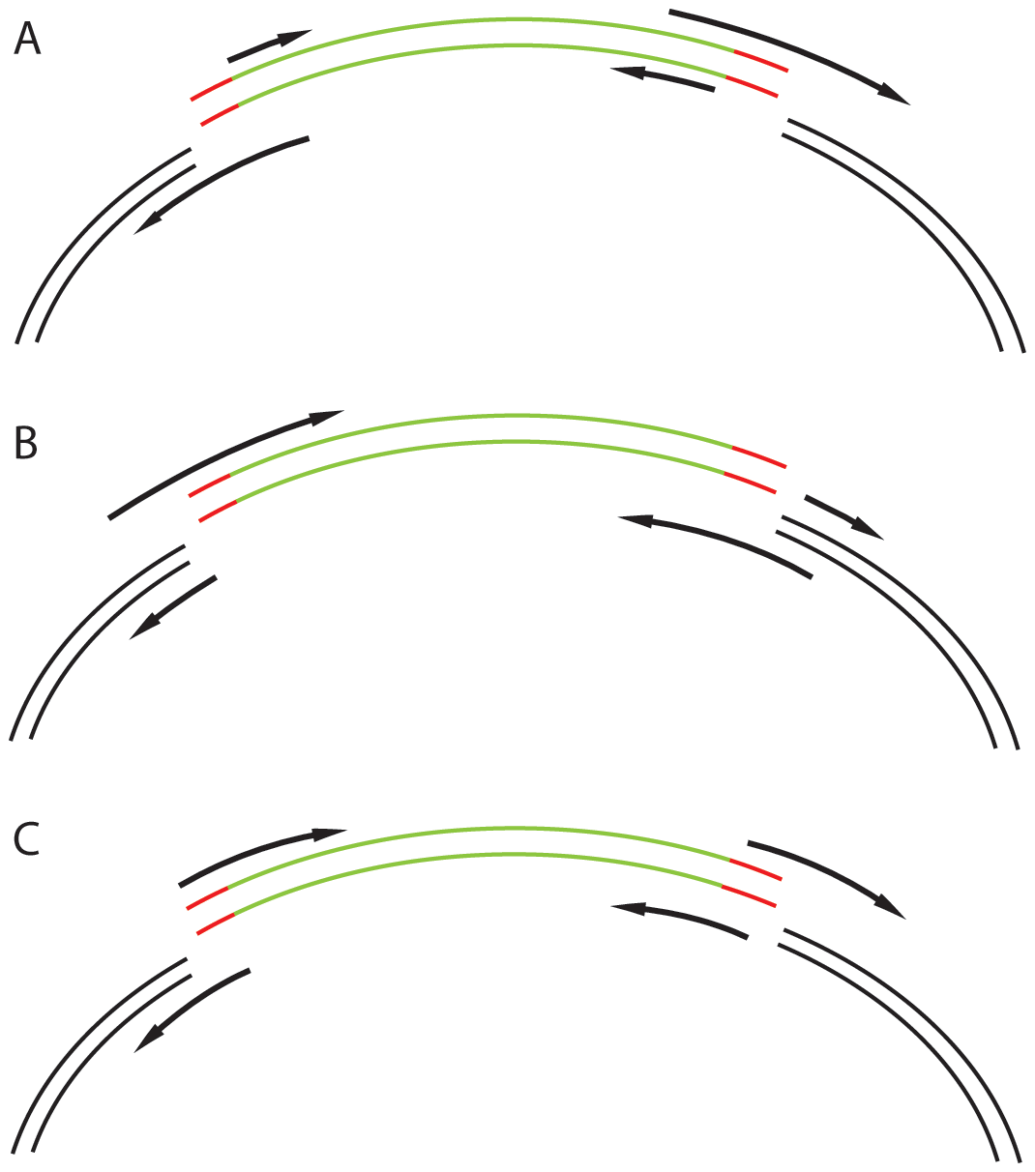
PCR cloning primer design software interface. (1) Upstream portion of the vector, up to the cloning position. (2) Additional sequence to include between the vector and the 5’ end of the target gene, eg protease cleavage site. (3) Sequence of the target, encoded by the template. (4) Additional sequence to include between the 3’ end of the target gene, eg protease cleavage site. (5) Downstream portion of the vector. (6) Resulting sequence, i.e. (1)+(2)+(3)+(4)+(5). (7) Output window containing information about primer design and primers to be ordered. pv5 is the **p**rimer annealing to the **5**′ end of the **v**ector, pi5 is **p**rimer annealing to the **5**′ end of the **i**nsert, pv3 and pi3 are **p**rimers annealing to the **3**′ end of the **v**ector and the **i**nsert, correspondingly. Several versions of primers to be ordered are designed, see **Figure 5** for more detailed.

The cloning primer design software **(Figure 4)** simplifies the task of designing such primers. The inputs of the software are the approximately 50 bp long vector sequences upstream and downstream of the insert, as well as the sequence of the insert. There is also a possibility to include additional sequences between the insert and the vector. Such short sequences will be included in the primers and may be useful for insertion or replacement of restriction sites, protease cleavage sites or purification tags.

**Figure 5:**
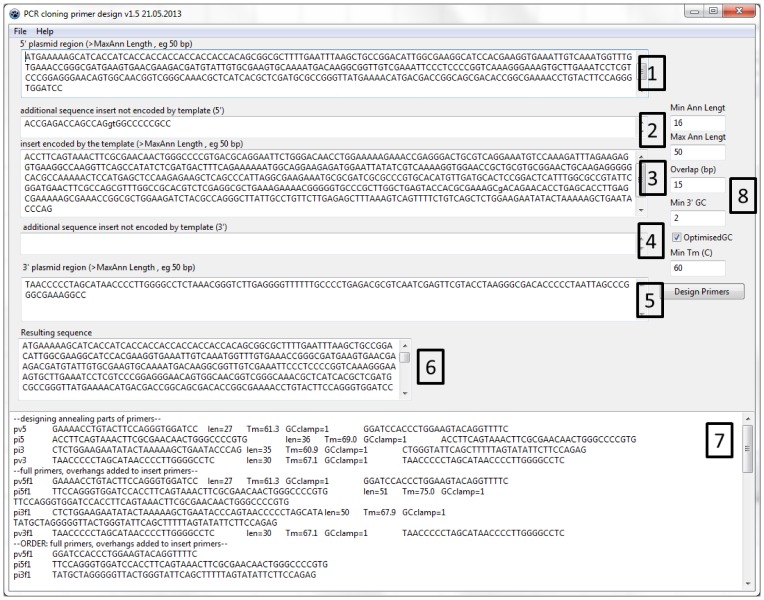
Various strategies for primer design. Green – insert, black – vector, red – additional optional sequences to be incorporated between the insert and the vector. Overhangs can be added to the vector-replicating primers (A), to the insert-replicating primers (B) or they can be split and added to both primers (C).

The software designs primers to amplify the vector and the insert. It subsequently adds the desired inclusions and the required 15 bp overlaps needed for homologous recombination. It offers the user several options of primer pair design which may be advantageous in different situations **(Figure 5)**. When cloning several different inserts into the same vector, it makes sense to have a primer pair for vector amplification and various primer pairs for amplification of the inserts. The opposite would be desired if the same insert was cloned into different vectors. In a third case, where due to the insertion of the relatively long additional DNA sequence the primers are long, and may be advantageous to have a balanced length of primers due to the technical difficulties of producing very long primers. In this case, four primers of balanced lengths would be advantageous when primers with long additional DNA sequences are designed.

## Experimental Results

### Optimisation of PCR conditions and workflow

We have optimised the PCR reaction conditions for obtaining single bands at 5-8 kb (linearized vectors) on 0.7% or 1% agarose gels. However, visually detectable bands were not essential to obtain mutant clones.

All solutions were strictly kept on ice. 1X Phusion® High-Fidelity PCR master mix with GC or HF buffer, or KOD polymerase with its supplied buffer, were supplemented with 400 mM TMSO and 12 pg/µL DNA template for amplification. 17 µl PCR master mix were combined with each 1.5 µL of both forward and reverse primer at stock concentration of 1 µM in each well of 96-well microplate.

For the PCR with a vector of approximately 8 kbp we used touchdown protocol [19], the detailed conditions were as follows: Initial denaturation at 98°C for 30 sec, then 20 step-down thermal cycles consisting of the denaturation at 98°C for 20 sec, annealing from 60°C down to 50°C for 30 sec with 0.5°C per cycle decrement, and extension at 72°C for 2min, followed by 20 thermal cycles (98°C for 20 sec, 54°C for 30 sec, 72°C for 2min), and final extension at 72°C for 5 min. Afterwards reactions were kept at 10°C.

DpnI digestion was also optimised to reduce the background. We have incubated 20 units of DpnI in 20 µL PCR mixture overnight at 37°C.

### Transformation and sequencing

**Figure 6:**
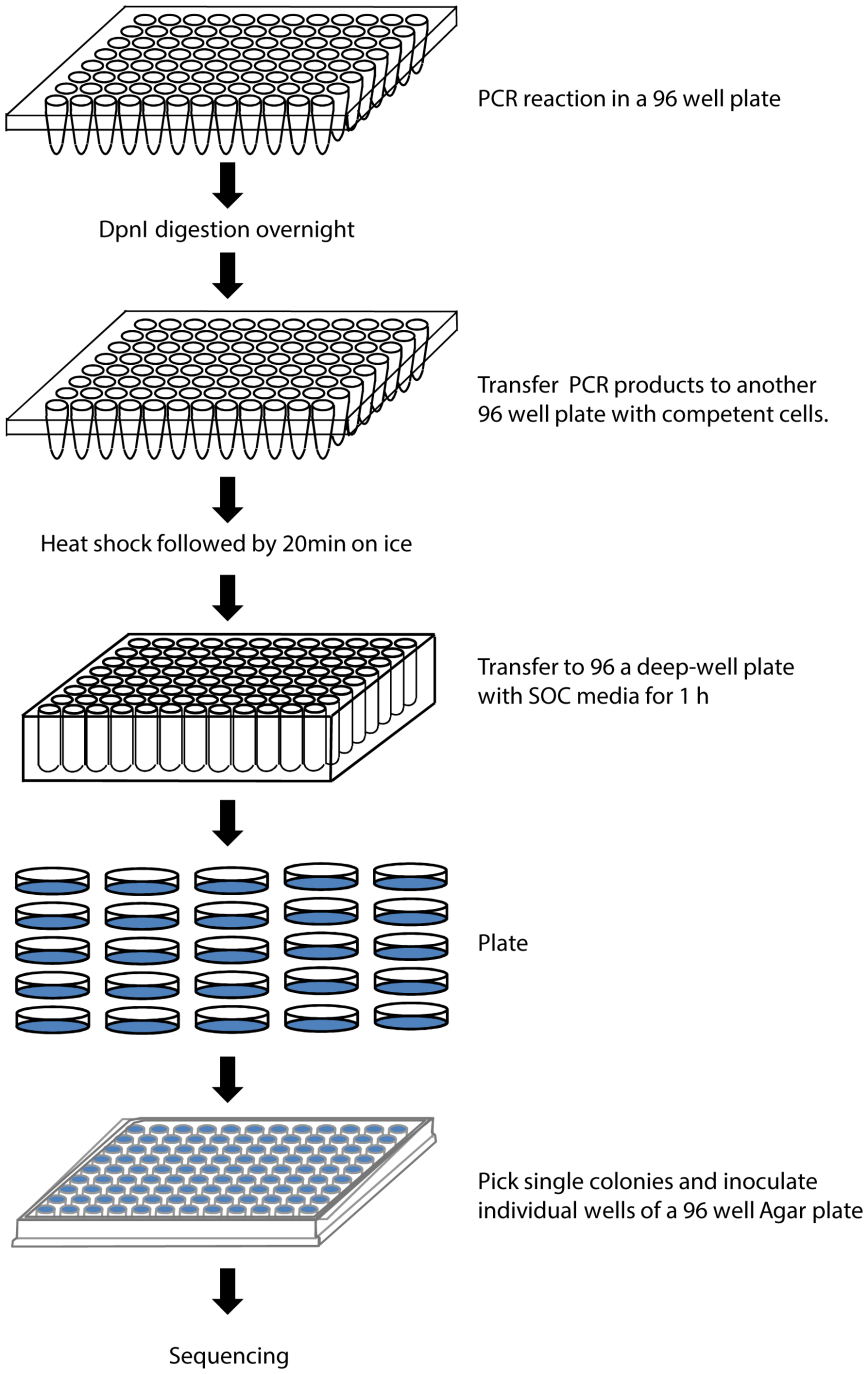
Workflow of the high throughput mutagenesis.

We have used the *E.coli* strain Mach1 for all DNA manipulations. The chemically competent cells had an efficiency of >10^7^ colonies/µg of pBR322 DNA for reliable results. Transformation steps were also optimized by adding 4 µl of PCR product into another 96-well microplate filled with 50 µl Mach1 cell suspension. After 25min on ice, the mixture was incubated in the PCR machine at 42°C (heat shock) for 45 s and then placed back on ice for 2min. The transformed cells were then transferred to a 96 deep-well plate filled with 600 µl S.O.C. media and incubated at 37°C for 2 h. 100 µl of 650 µl samples were plated on LB agar plate with appropriate antibiotics and incubated at 37°C overnight. A single colony of each mutant was transferred into a 96 well Agar plate with appropriate antibiotics and sequenced by the GATC Biotech Company **(Figure 6)**.

To minimise sequencing costs, we have sent one or two clones for sequencing, and sent additional ones only if the first round of sequencing did not yield the desired mutation. It has been relatively easy to achieve 80% success rate – on average only about two colonies for each mutant had to be sequenced. Sending more clones for sequencing yields missing mutants, however some proved to be very difficult to obtain. If repeating the whole mutagenesis procedure with alternative polymerase still did not produce the desired mutant, we resorted to alternative mutagenesis strategies.

Of 403 mutants in arrestin we obtained 297 (74% coverage) in the 1^st^ round, 42 (84%) in the 2^nd^, 12 (87%) in the 3^rd^ and 13 (90%) in the 4^th^ round of sequencing one clone of a mutant. In three subsequent rounds we obtained another 18 mutants by using PCR products cleaned up by the ExoSapIT kit, and using alternative primers, also suggested by AAscan. The remaining 21 mutants were constructed by the two fragment approach (see below). In the case of Galphai1, we obtained 289 out of 354 mutants (82%) in three rounds of mutagenesis. The remaining 65 mutants were ordered as synthetic constructs.

The most common problem we have observed at the sequencing level, was insertion of tandem repeats of the mutagenesis primer at the mutation site [20]. Reducing the amount of template DNA to 0.2 ng seems to minimise this artefact. The second commonly observed problem was deletion of a part of a sequence, either upstream or downstream of the mutation site. Both problems are easily detectable by mutant checker software or by manual alignment of the sequencing results to the reference sequence. We have observed that the “problem” mutants tend to cluster, and in several cases there was a stretch of amino acid positions for which we were not able to obtain alanine mutants by this technique. As the primers generated for the neighbouring positions have to a large degree identical sequence, it is possible that certain sequence related features lead to undesired side products of the mutagenic PCR.

### Effect of primer properties on success of mutagenesis

**Figure 7:**
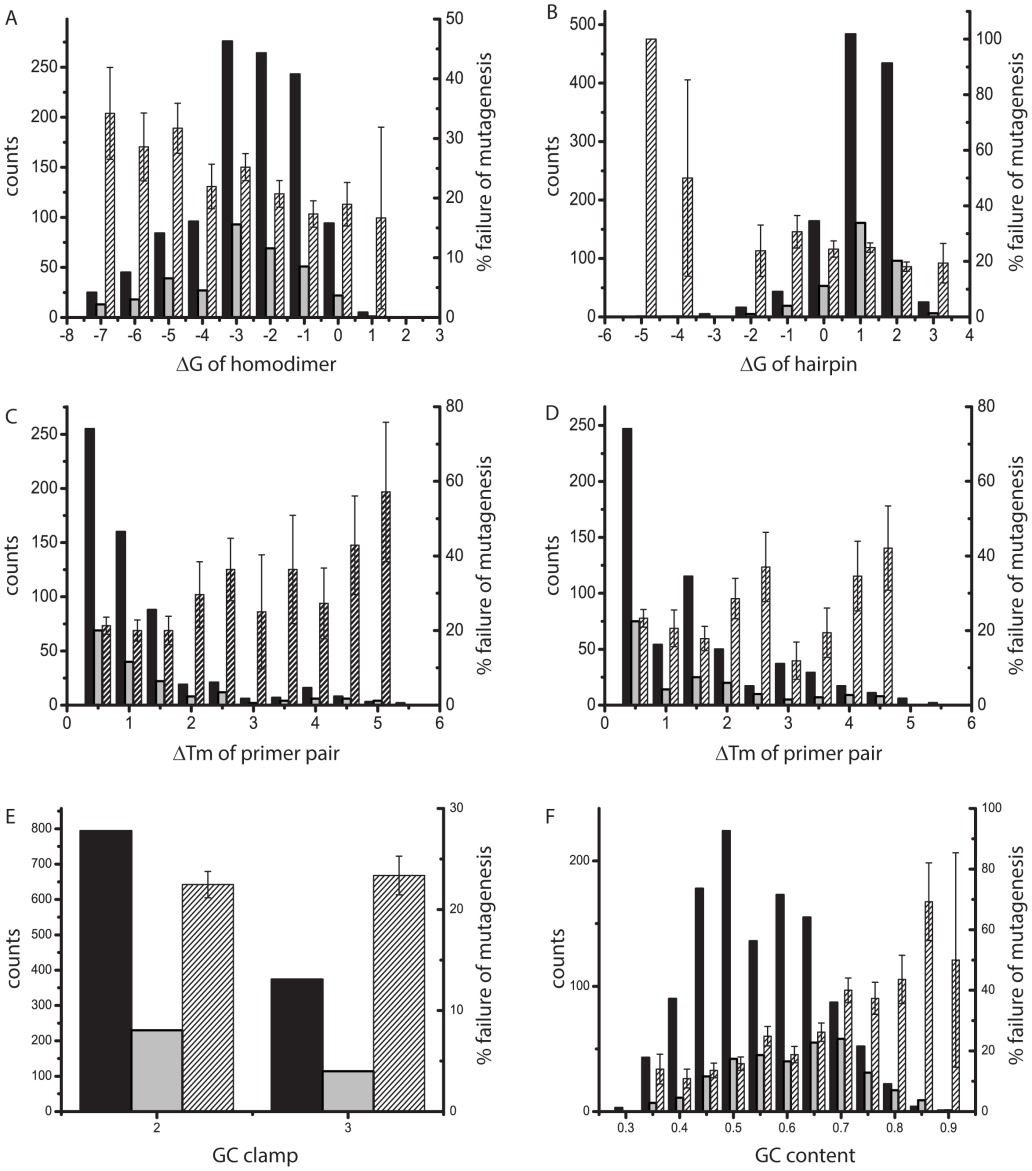
Influence of secondary structure and melting temperatures of primers on the success of mutagenesis. Features of primer pairs (ΔTm) are counted once, features of single primers (ΔG) were counted separately for the reverse and forward primer of a pair and are represented in one graph. Solid bars represent the number of primers with a particular value of a feature (left axis), striped bar represent the fraction failed (right axis). The standard deviation of the fraction failed was calculated as (f*(1-*f*)/*N*)^1/2^, where *f* is fraction failed and *N* is the total number of primers in a particular category. Success (black) or failure (grey) of mutagenesis, as well as fraction failed (striped) in dependence of **(A)** the ΔG of hairpins formed by the primers, **(B)** the ΔG of homodimers formed by the primers, **(C)** primer melting temperatures calculated for mutation of the native DNA (early PCR cycles), **(D)** primer melting temperatures calculated for mutation of the DNA containing the mutation (later cycles of PCR), **(F)** quality score of the GC clamp and **(G)** GC content of the primer.

Here, we evaluate whether the success of mutagenesis depends on the thermodynamic stability of the primers used to generate the mutations. We used data from alanine scans of two complete proteins, arrestin, 404 residues, and Galphai1, 354 residues long. The primers generated by the AAscan program were used for mutagenesis as described above, introducing one alanine or glycine mutation at a time. Mutations were classified as successful if the mutation was obtained with this technique using the first set of primers given by the program. It is generally assumed that secondary structure of the primer, notably homodimers and hairpins, may interfere with the efficiency of the PCR. The Gibbs energy of the most stable homodimer and hairpin, respectively, as well as the thermodynamic melting temperature (T_m_), which depends on the GC content of the primer, was calculated for all 758 forward and reverse pairs of primers **(Figure 7)**.

Our data indicate that stable secondary structures and higher differences in melting temperatures of the primers do not impair the probability of inserting the mutation. The success rate for primers with stable secondary structure is very similar to the overall success rate. However, there may be a weak correlation between increase in stability of secondary structure and the rate of failure (R factor of 0.76 for homodimer and 0.61 for the hairpin formation). The difference in the Tm of the primers seems to have little if any correlation with the failure rate (R factors of 0.52 and -0.19 for early and late PCR stages). A quality of the GC clamp (see above) does not affect the success of mutagenesis, while increase in the GC content of the primers is weakly correlated with increase failure rate(R = 0.77). Overall, there was no strong correlation between the secondary structure, Tm or GC content of the primers and the failure of the mutagenic PCR.

### A two-fragment mutagenesis approach

**Figure 8:**
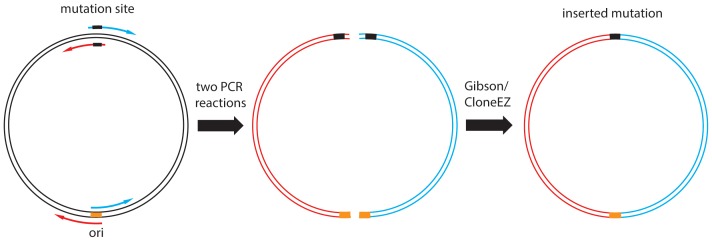
Two-fragment PCR mutagenesis strategy for difficult mutants. Each mutagenesis primer is used together with another primer annealing approximately opposite the mutation site, e.g. origin region of the plasmid. The resulting two PCR fragments are re-combined by CloneEZ or Gibson reaction to form the original circular plasmid.

To generate the mutants not obtained by the method described above we used an alternative two-fragment approach. Each mutagenic primer was used for a PCR together with a suitable primer on the complementary strand in the origin region of the plasmid **(Figure 8)**. While potentially any pair of primers could be chosen, the origin region was approximately on the opposite side of the plasmid from the mutagenesis site. This way we generated two PCR products of about half the size of the plasmid. In addition, the origin region is identical in many expression vectors, making it a convenient choice. After checking their size on a gel, the fragments were purified by reaction cleanup (Qiagen) and assembled by the CloneEZ reaction (Genscript) or by the Gibson reaction [21]. This approach is more laborious, but it was successful in all 15 cases tried.

## Conclusions

Making mutants by a PCR method using primers designed by AAscan software has a success rate similar to other reported studies. Of course, the task of designing primers is very much simplified. The biggest advantage is the automatic design of a series of primers, which eliminates the errors associated with one by one, and the time savings offered. There is a certain fraction of mutants which we were not able to obtain using this method, and it is most efficient to use alternative methods in these cases, such as the two fragment approach described above, or synthetic DNA constructs. The open source nature of the AAscan and associated software makes it easily adaptable to various scenarios. We hope that AAscan and associated software will become a useful tool for many laboratories.

## Materials and Methods

All primers were ordered from Intergrated DNA technologies, as desalted oligonucleotides, on a 96-well plate, concentration normalised to 100 µM. Phusion High-Fidelity PCR master mix with HF and GC buffer was purchased from Thermal Scientific. DpnI was purchased from Fermentas. ExoSapIT was from Affymetrix. All PCRs were performed using Eppendorf Mastercycler pro S. The S.O.C media contains 2% (w/v) Tryptone, 0.5% (w/v) yeast extract, 8.6 mM NaCl, 2.5 mM KCl, 20 mM MgSO_4_, and 20 mM Glucose. Sequencing was done by GATC Biotech (Germany).

Thermodynamic properties of primers were determined using the VectorNTI® Advance 11.5 software (Invitrogen) and the OligoArrayAux programme from the UNAfold software package (Markam and Zuker, 2008). The calculations of thermodynamic properties by VectorNTI® Advance 11.5 and OligoArrayAux were compared at 25°C and a salt concentration of 50 mM. The nucleic acid type was set to DNA. Otherwise, the default settings of the two programs were used.

The OligoArrayAux hybrid-ss-min and hybrid-min programmes were used for the calculation of Gibbs energies for primer hairpins and homodimers. The temperature was set to 65°C as this temperature can be used for annealing of the primers in PCR. The calculated Gibbs energy is given in kcal/mol.

### Software development and availability

Software was developed using FreePascal and Lazarus IDE (http://www.lazarus.freepascal.org/).

Precompiled executable files and a source code are available at http://www.psi.ch/lbr/alascan.
